# Performance of blood concentrates in controlling inflammatory signs and symptoms after lower third molar extractions: an overview

**DOI:** 10.1590/acb401825

**Published:** 2025-03-10

**Authors:** Vinícius Lima de Almeida, Marcelo Dias Moreira de Assis Costa, Caio Melo Mesquita, Walbert Andrade Vieira, Rafael Rodrigues Lima, Livia Bonjardim Lima, Sigmar de Mello Rode, Luiz Renato Paranhos

**Affiliations:** 1 Universidade Federal de Uberlândia – School of Dentistry – Post-Graduation Program in Dentistry – Uberlândia (MG) – Brazil.; 2 Universidade Federal de Minas Gerais – School of Dentistry – Department of Clinics, Pathology, and Oral Surgery – Belo Horizonte (MG) – Brazil.; 3 Universidade Federal de Uberlândia – School of Medicine – Multiprofessional Health Residency Program in Critical Patient Care – Uberlândia (MG) – Brazil.; 4 Centro Universitário das Faculdades Associadas de Ensino – Department of Dentistry – São João da Boa Vista (SP) – Brazil.; 5 Universidade Federal do Pará – Institute of Biological Sciences – Laboratory of Functional and Structural Biology – Belém (PA) – Brazil; 6 Universidade Federal de Uberlândia – School of Dentistry, Department of Oral and Maxillofacial Surgery and Traumatology – Uberlândia (MG) – Brazil.; 7 Universidade Estadual Paulista – Institute of Science and Technology – Department of Dental Materials and Prothesis – São José dos Campos (SP) – Brazil.; 8 Universidade Federal de Uberlândia – School of Dentistry – Department of Preventive and Community Dentistry – Uberlândia (MG) – Brazil.

**Keywords:** Systematic Review, Inflammation, Platelet-Rich Fibrin, Platelet-Rich Plasma

## Abstract

**Purpose:**

To summarize the available evidence and answer the following question: What is the current knowledge on the performance of blood concentrates in handling sequelae after lower third molar extractions with the evidence available in systematic reviews?

**Methods:**

An electronic search was conducted across nine databases. The study included systematic reviews with or without meta-analyses investigating the performance of blood concentrates in managing sequelae after lower third molar extractions. The four outcomes analyzed were pain, edema, mouth opening, and alveolar osteitis. The AMSTAR-2 tool assessed the methodological quality of the included systematic reviews, while ROBIS evaluated the risk of bias.

**Results:**

The electronic search revealed 690 records, of which 15 were eligible systematic reviews for the present study. Overall, these reviews evaluated 75 primary studies published from 2007 to 2023. According to AMSTAR-2, only one systematic review presented high methodological quality. The ROBIS tool showed two systematic reviews with a low risk, and the others had a high risk of bias.

**Conclusion:**

The current evidence is based on only one systematic review with high methodological quality and a low risk of bias, while the others exhibited a high risk of bias and low methodological quality. Therefore, the evidence regarding the efficacy of blood concentrates in controlling sequelae following lower third molar extractions is inconclusive.

## Introduction

Lower third molar extractions may be associated with sequelae, such as pain, edema, mouth-opening difficulties, and alveolar osteitis, harming patients’ postoperative quality of life^1^^,^ 2. Several therapeutic approaches have been indicated to control these inflammatory signs and symptoms, including drug therapy, cryotherapy, low-level laser therapy, and blood concentrates^2^^–^ 7.

Blood concentrates originate from autologous blood and primarily consist of platelets, fibrin, leukocytes, and growth factors^8^. Platelet-rich plasma (PRP) is the first-generation blood concentrate, presenting disadvantages such as the need for more than one step for production and anti- and procoagulant addition^9^^,^ 10. Studies have developed blood products, such as platelet-rich fibrin (PRF), later renamed leukocyte- and platelet-rich fibrin (L-PRF)^11^. New centrifugation protocols developed from L-PRF resulted in advanced platelet-rich fibrin (A-PRF), advanced platelet-rich fibrin plus (A-PRF+), and concentrated growth factor (CGF)^10^^–^ 12.

Blood concentrates are fibrin-based biomaterials containing cells, matrix proteins, pro- and anti-inflammatory mediators, and growth factors, showing positive performance in handling postoperative sequelae, such as pain, edema, trismus, and alveolar osteitis^13^^,^ 14. Hence, inflammatory response modulation occurs, optimizing primary hemostasis, chemotaxis, angiogenesis, and mitogenesis of endothelial cells^14^.

Systematic reviews have analyzed the effect of blood concentrates on managing postoperative sequelae, such as pain, edema, mouth opening, and alveolar osteitis, showing conflicting results and several recommendations for randomized clinical trials with larger samples and more homogeneous methodologies^15^^–^ 18. Moreover, the overall syntheses and assessments of these reviews have not occurred, which is relevant considering each review answers different research questions on the same subject. These reviews analyze different primary studies and blood concentrates, causing the referred outcome variability. Therefore, this overview summarized the available evidence and answered the following question: What is the current knowledge on the performance of blood concentrates in handling sequelae after lower third molar extractions with the evidence available in systematic reviews?

## Methods

### Protocol registration

The protocol of this review was created according to the PRISMA-P^19^ guidelines and registered *a priori* in the PROSPERO database (CRD42023405301) (https://www.crd.york.ac.uk/prospero/). The overview was reported following the Preferred Reporting Items for Systematic Reviews and Meta-Analyses (PRISMA) recommendations^20^.

### Eligibility criteria

The study included systematic reviews with or without meta-analyses investigating the performance of blood concentrates in controlling sequelae after lower third molar extractions. The four outcomes analyzed were pain, edema, mouth opening, and alveolar osteitis. There were no restrictions on year or language.

The exclusion criteria were:

- Randomized clinical trials;
- Studies with different objectives;
- Case reports;
- Animal studies;
- *In-vitro* studies;
- Letters to the editor;
- Observational studies;
- Systematic reviews with or without meta-analyses and with outcomes not directly related to pain, edema, mouth opening, and alveolar osteitis.

### Sources of information, search, and selection of studies

The electronic searches were performed in June 2022 and updated in May 2024 in the Cochrane Library, Embase, Latin American and Caribbean Health Sciences Literature (LILACS), MEDLINE (via PubMed), Scientific Electronic Library Online (SciELO) databases, and Scopus and Web of Science citation databases. The EASY and the MedRxiv preprint databases partially captured the grey literature. A search strategy was formulated for each database, respecting their syntax rules and using the Health Science Descriptors (DeCS), Medical Subject Headings (MeSH), and Embase Subject Headings (Emtree) descriptors with the Boolean operators AND/OR to maximize the search. Table 1 describes the search strategy for each database.

**Table 1:** Search strategies.

Databases	Search strategies (June 2022) and Update (May 2024)
Primary databases	
Cochrane Library https://www.cochranelibrary.com/	#1 «Molar, Third» OR «Wisdom Tooth» OR «Wisdom Teeth» OR «Third Molar» in All Text #2 «Pain, Postoperative» OR «Pain» OR «Edema» OR «Swelling» OR «Trismus» OR «Dry Socket» OR «Alveolar Osteitis» OR «Wound Healing» OR «Wound» OR «Healing» OR «Recovery» OR «Morbidity» OR «Outcomes» OR «Efficacy» OR «Comparison» in All Text #3 «Platelet-Rich Fibrin» OR «PRF» OR «Platelet-Rich» in All Text
	#1 AND #2 AND #3
Embase https://www.embase.com	(‹molar, third›/exp OR ‹molar, third› OR ‹wisdom tooth›/exp OR ‹wisdom tooth› OR ‹wisdom teeth›/exp OR ‹wisdom teeth› OR ‹third molar›/exp OR ‹third molar›) AND (‹pain, postoperative›/exp OR ‹pain, postoperative› OR ‹pain›/exp OR ‹pain› OR ‹edema›/exp OR ‹edema› OR ‹swelling›/exp OR ‹swelling› OR ‹trismus›/exp OR ‹trismus› OR ‹dry socket›/exp OR ‹dry socket› OR ‹alveolar osteitis›/exp OR ‹alveolar osteitis› OR ‹wound healing›/exp OR ‹wound healing› OR ‹wound›/exp OR ‹wound› OR ‹healing›/exp OR ‹healing› OR ‹recovery›/exp OR ‹recovery› OR ‹morbidity›/exp OR ‹morbidity› OR ‹outcomes›/exp OR ‹outcomes› OR ‹efficacy›/exp OR ‹efficacy› OR ‹comparison›/exp OR ‹comparison›) AND (‹platelet-rich fibrin›/exp OR ‹platelet-rich fibrin› OR ‹prf› OR ‹platelet-rich›)
LILACS http://pesquisa.bvsalud.org/	((“Molar, Third” OR “Wisdom Tooth” OR “Wisdom Teeth” OR “Third Molar”) AND (“Pain, Postoperative” OR “Pain” OR “Edema” OR “Swelling” OR “Trismus” OR “Dry Socket” OR “Alveolar Osteitis” OR “Wound Healing” OR “Wound” OR “Healing” OR “Recovery” OR “Morbidity” OR “Outcomes” OR “Efficacy” OR “Comparison”) AND (“Platelet-Rich Fibrin” OR “PRF” OR “Platelet-Rich”)) AND ( db:(“LILACS”))
MEDLINE (*via* PubMed) http://www.ncbi.nlm.nih.gov/pubmed	#1 “Molar, Third”[Mesh] OR “Wisdom Tooth”[tw] OR “Wisdom Teeth”[tw] OR “Third Molar”[tw] #2 “Pain, Postoperative”[Mesh] OR “Pain”[Mesh] OR “Edema”[Mesh] OR “Swelling”[tw] OR “Trismus”[Mesh] OR “Dry Socket”[Mesh] OR “Alveolar Osteitis”[tw] OR “Wound Healing”[Mesh] OR “Wound”[tw] OR “Healing”[tw] OR “Recovery”[tw] OR “Morbidity”[Mesh] OR “Outcomes”[tw] OR “Efficacy”[tw] OR “Comparison”[tw] #3 “Platelet-Rich Fibrin”[Mesh] OR “PRF”[tw] OR “Platelet-Rich”[tw]
	#1 AND #2 AND #3
SciELO https://scielo.org/	((“Molar, Third” OR “Wisdom Tooth” OR “Wisdom Teeth” OR “Third Molar”) AND (“Pain, Postoperative” OR “Pain” OR “Edema” OR “Swelling” OR “Trismus” OR “Dry Socket” OR “Alveolar Osteitis” OR “Wound Healing” OR “Wound” OR “Healing” OR “Recovery” OR “Morbidity” OR “Outcomes” OR “Efficacy” OR “Comparison”) AND (“Platelet-Rich Fibrin” OR “PRF” OR “Platelet-Rich”))
Scopus http://www.scopus.com/	((“Molar, Third” OR “Wisdom Tooth” OR “Wisdom Teeth” OR “Third Molar”) AND (“Pain, Postoperative” OR “Pain” OR “Edema” OR “Swelling” OR “Trismus” OR “Dry Socket” OR “Alveolar Osteitis” OR “Wound Healing” OR “Wound” OR “Healing” OR “Recovery” OR “Morbidity” OR “Outcomes” OR “Efficacy” OR “Comparison”) AND (“Platelet-Rich Fibrin” OR “PRF” OR “Platelet-Rich”)
Web of Science http://apps.webofknowledge.com/	#1 TS=(“Molar, Third” OR “Wisdom Tooth” OR “Wisdom Teeth” OR “Third Molar”) #2 TS=(“Pain, Postoperative” OR “Pain” OR “Edema” OR “Swelling” OR “Trismus” OR “Dry Socket” OR “Alveolar Osteitis” OR “Wound Healing” OR “Wound” OR “Healing” OR “Recovery” OR “Morbidity” OR “Outcomes” OR “Efficacy” OR “Comparison”) #3 TS=(“Platelet-Rich Fibrin” OR “PRF” OR “Platelet-Rich”)
	#1 AND #2 AND #3
**Grey literature**	
EASY https://easy.dans.knaw.nl/	(«Third molar» OR «Platelet-Rich Fibrin» OR «PRF» OR «Platelet-Rich»)
MedRxiv https://www.medrxiv.org/	((«Molar, Third» OR «Wisdom Tooth» OR «Wisdom Teeth» OR «Third Molar») AND («Platelet-Rich Fibrin» OR «PRF» OR «Platelet-Rich»))

Source: Elaborated by the authors.

The search results were exported to EndNote Web^TM^ software (Thomson Reuters, Toronto, ON, Canada) to identify and remove duplicates. Next, they were exported to Rayyan QCRI software (Qatar Computing Research Institute, Doha, Qatar) for title and abstract analyses according to the described eligibility criteria. Subsequently, the full texts of the preliminary eligible studies were obtained and evaluated. Microsoft Word 2010 (Microsoft, Redmond, WA, United States of America) assisted in simultaneously evaluating grey literature results. Two eligibility reviewers (VLA and CMM) performed this entire process independently. Divergences were solved after consulting with a third eligibility reviewer (LRP).

### Data collection

After selecting the articles, two reviewers (VLA and CMM) extracted the following information: author, year, and journal of publications; research objective; blood concentrate type; searched databases and period; primary studies included in the systematic reviews; risk of bias assessment tools of the studies included in the systematic reviews; and primary results and conclusions. The reviewers (VLA and CMM) underwent a calibration exercise to ensure consistency during data extraction by jointly extracting information from an eligible study. In case of missing data, the corresponding authors of the eligible studies were contacted via e-mail, with three weekly contact attempts for up to a month.

### Critical assessment of individual evidence sources

Three reviewers (VLA, CMM, and LRP) analyzed the methodological quality of the included systematic reviews using the AMSTAR-2 critical assessment tool^21^. This tool contains 16 questions (seven critical and nine non-critical), and their answer options were “yes,” “partially yes,” “no,” or “no meta-analysis performed.” The methodological quality was low if one critical question had a “no” response and critically low if two or more critical questions had “no” answers.

### Risk of individual bias in the studies

Three reviewers (VLA, CMM, and LRP) independently assessed the risk of bias in the included systematic reviews using the ROBIS tool for evaluating the risk of bias in systematic reviews^22^.

Initially, they analyzed four domains from which biases might have been introduced in a systematic review:

- Eligibility criteria;
- Identification and selection of articles;
- Data collection and study assessment;
- Synthesis and results.

Moreover, each domain has five or six signaling questions with the following possible answers: “yes (Y),” “probably yes (PY),” “probably no (PN),” “no (N),” “not informed (NI),” or “not applicable (NA).” Later, the overall risk of bias was evaluated during the interpretation of review findings, considering whether it identified limitations within the described domains.

All reviewers agreed on the decisions about the scoring system and cutoffs before analyzing biases. The authors determined the classification system for the bias of each domain as a “low risk” if all signaling questions scored Y/PY, “unclear risk” if only one question received PN/N/NI, and “high risk” if more than one question scored PN/N/NI. Moreover, the overall risk of bias regarding each systematic review was low if all four domains had a “low risk” or only one had an “unclear risk,” moderate if two or more domains had an “unclear risk,” and high if one or more domains had a “high risk.”

## Results

### Selection of systematic reviews

**Figure 1:**
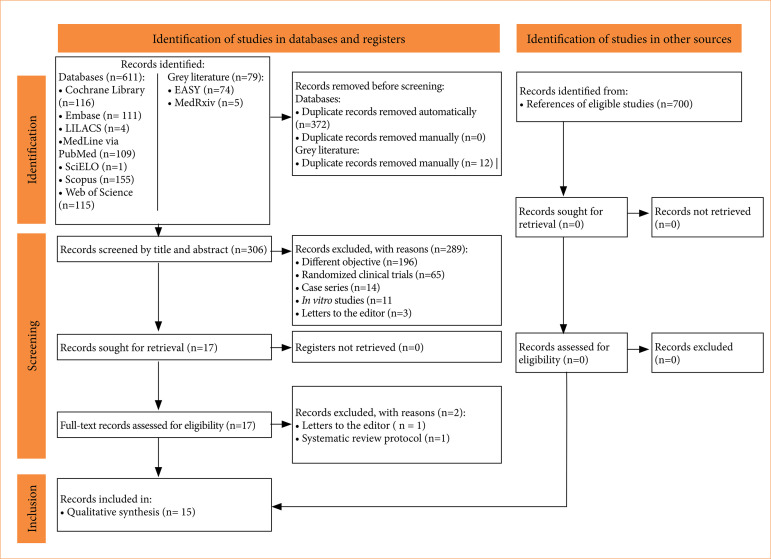
Flowchart showing the selection process of the eligible studies.

Source: Elaborated by the authors.

The study identification process found 690 records. After removing duplicates and reading the titles and abstracts, only 17 records were fully assessed for eligibility criteria, of which two were eliminated. Finally, 15 systematic reviews were selected^15^^–^ 18 ^,^ 23 ^–^ 33. The list of references of the eligible studies were carefully searched but did not reveal additional systematic reviews. Figure 1 details the study selection in a flowchart.

### Characteristics of the included systematic reviews

The analysis comprised systematic reviews published between 2007 and 2024. One study was published in Chinese^28^, and the others in English. There were similarities in the search databases, with all reviews including MEDLINE (via PubMed). Additionally, seven systematic reviews manually searched journals or the list of references of their primary studies^16^^,^ 17 ^,^ 23 ^,^ 24 ^,^ 29 ^,^ 31 ^,^ 32. Table 2 describes the main characteristics of the included systematic reviews.

**Table 2:** Main characteristics of the included systematic reviews.

Authors, year journal	Objectives	Blood concentrate	Databases (Electronic search date)	Number of included primary studies		Risk of bias tool
				Qualitative synthesis	Quantitative synthesis	
Al-Hamed et al. (2017)^23^ *J Oral Maxillofac Surg*	To assess the effect of PRF on socket healing after the surgical extraction of lower third molars.	PRF	**Main databases:** Cochrane Central Register of Controlled Trials; PubMed; Scopus. (August/November 2015)	6	- AO (n = 1) - BR (n = 2) - Trismus (n = 2)	Cochrane Collaboration tool
Bao et al. (2021)^27^ *J Oral Maxillofac Surg*	To investigate the use of L-PRF and A-PRF for relieving signs and symptoms after lower third molar extraction.	A-PRF L-PRF	**Main databases:** Embase; PubMed; SinoMed; Web of Science. (October 2020)	10	- AO (n = 2) - Pain (n = 6) - WH (n = 4)	Cochrane Collaboration tool
Bao et al. (2021)^28^ *West China J Stomatol*	To assess the efficacy of PRF in relieving complications after lower third molar extraction.	PRF	**Main databases:** Embase; PubMed; SinoMed; Web of Science. (February 2020)	21	- AC (n = 2) - AO (n = 3) - BR (n = 2) - Edema (n = 5) - Pain (n = 6) - Trismus (n = 2) - WH (n = 3)	Cochrane Collaboration tool
Barona-Dorado et al. (2014)^15^ *Med Oral Patol Oral Cir Bucal*	To assess the scientific evidence supporting PRP application in post-extraction sockets of retained lower third molars.	PRP	**Main databases:** Cochrane Central Register of Controlled Trials; Embase via Ovid; MEDLINE via PubMed; NIH. (June 2013)	3	Meta-analysis was not performed.	Jadad scale
Canellas et al. (2017)^16^ *Int J Oral Maxillofac Surg*	To assess potential differences in postoperative complications when using PRF in mandibular third molar surgeries.	PRF	**Main databases:** Cochrane Library; LILACS; MEDLINE via PubMed; ScienceDirect. **Secondary databases:** Theses and Dissertations Base (CAPES); Current Controlled Trials; ClinicalTrials.gov; EU Clinical Trials Register. (August 2016)	7	- AO (n = 2)	Cochrane Collaboration tool
Canellas et al. (2019)^25^ *Int J Oral Maxillofac Surg*	To assess to which indications PRF has been effective in oral surgical procedures.	PRF	**Main databases:** Cochrane Library; LILACS; MEDLINE via PubMed; Scopus; Web of Science. **Secondary databases:** ClinicalTrials.gov; EU Clinical Trials Register. (September 2017)	30	- AO (n = 3) - BR (n = 2) - BRMSL (n = 2) - DIS (n = 2) - Edema (n = 3) - Pain (n = 6)	Cochrane Collaboration tool
Costa et al. (2023)^32^ *Clin Oral Investig*	To evaluate the effectiveness of different blood concentrates in controlling inflammatory signs and symptoms after lower third molars extractions.	PRP A-PRF L-PRF CGF	**Main databases:** Cochrane Library; Web of Science; Embase; MEDLINE via PubMed; Scopus; Lilacs; SciELO. **Secondary databases:** OpenGrey; MedRxiv. (March 2022 / May 2023)	36	- Pain (n = 18) - Trismus (n = 5)	Cochrane Collaboration tool
Fujioka-Kobayashi et al. (2021)^17^ *J Oral Maxillofac Surg Med Pathol*	To investigate the efficacy of PRF in handling lower third molar extractions from randomized clinical trials.	PRF	**Main databases:** Cochrane Central Register of Controlled Trials; Embase; LILACS; MEDLINE via PubMed; Scopus. **Secondary databases:** Grey Literature Report; OpenGrey. (June 2020)	18	- AO (n = 5)	RoB 2
He et al. (2017)^24^ *J Oral Maxillofac Surg*	To analyze the local PRF application to control postoperative signs and symptoms after extracting an impacted lower third molar.	PRF	**Main databases:** Cochrane Library; Embase; PubMed; Web of Science. (October 2016)	10	- AO (n = 4) - BR (n = 2) - Edema (n = 3) - Pain (n = 6) - Trismus (n = 3)	Cochrane Collaboration tool
Ramos et al. (2022)^30^ *J Oral Maxillofac Surg*	To determine whether different platelet aggregation protocols (PRF/L-PRF and A-PRF) have similar effects on pain, edema, and trismus control.	PRF A-PRF L-PRF	**Main databases:** Cochrane; Embase; MEDLINE; PubMed; BVS; Web of Science. (April/July 2021)	17	- Edema (n = 5) - Pain (n = 7) - Trismus (n = 5) - WH (n = 5)	Cochrane Collaboration tool
Ribeiro et al. (2024)^33^ *Clin Oral Investig*	L-PRF		**Main databases:** Cochrane Library; Web of Science; Embase; MEDLINE via PubMed; Scopus. (December 2023)	5	Meta-analysis was not performed.	Cochrane Collaboration tool
Snopek et al. (2022)^31^ *Oral Surg*	PRF		**Main databases:** Cochrane Library; Embase; MEDLINE via PubMed; Scopus; Web of Science. **Secondary databases:** ClinicalTrials.gov; Grey Literature Report; OpenGrey. (June 2020)	6	- Pain (n = 3)	Cochrane Collaboration tool
Vitenson et al. (2022)^18^ *Int J Oral Maxillofac Surg*	A-PRF L-PRF		**Main databases:** Cochrane Library; Embase; MEDLINE via PubMed; Scopus. **Secondary databases:** OpenGrey; OpenThesis. (December 2020)	4	- Edema (n = 3) - Pain (n = 3) - Trismus (n = 3)	Cochrane Collaboration tool
Xiang et al. (2019)^26^ *BMC Oral Health*	PRF		**Main databases:** Cochrane Library; Embase; PubMed. (September 2017)	10	- AO (n = 3) - BR (n = 2) - Edema (n = 4) - Pain (n = 6) - Trismus (n = 4) - WH (n = 2)	Cochrane Collaboration tool
Zhu et al. (2021)^29^ *Int J Oral Maxillofac Surg*	PRF		**Main databases:** Cochrane Library; Embase; PubMed; Web of Science. (May 2019)	19	- AO (n = 6) - Pain (n = 13) - Trismus (n = 3) - WH (n = 4)	Cochrane Collaboration tool

PRP: platelet-rich plasma; PRF: platelet-rich fibrin; A-PRF: advanced platelet-rich fibrin; L-PRF: leukocyte- and platelet-rich fibrin; CGF: concentrated growth factor; AO: alveolar osteitis; BR: bone repair; DIS: dental implant stability; BRMSL: bone repair in maxillary sinus lift; WH: wound healing; AC: analgesic consumption; NIH: National Institutes of Health; LILACS: Latin American and Caribbean Health Sciences Literature; CAPES: Coordination of Superior Level Staff Improvement. Source: Elaborated by the authors.

**Figure 2:**
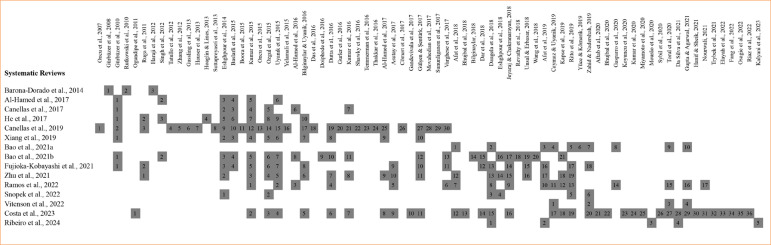
Overlap graphic.

Source: Elaborated by the authors.

These reviews evaluated 75 primary studies published between 2007 and 2023, with 32 common studies across multiple systematic reviews. Figure 2 illustrates the overlap of primary studies.

Considering the heterogeneity level regarding primary studies’ designs (parallel or split mouth), samples, measurement methods, and differences in the postoperative assessment of pain, edema, and trismus, two systematic reviews could not perform a meta-analysis for these outcomes^15^^,^ 33. Table 3 describes the main results of the systematic reviews.

**Table 3:** Main outcomes of the included systematic reviews.

Authors, year	Main results	Main conclusions
Al-Hamed et al. (2017)^23^	PRF showed overall positive results for pain, trismus, edema, periodontal pocket depth, soft tissue healing, and localized osteitis incidence. The meta-analysis could not be performed for all variables. The meta-analyses did not show significant benefits for PRF compared to natural healing sockets.	Considering the limitations of the available evidence, PRF does not seem beneficial for bone consolidation after lower third molar extractions. Further controlled and standardized randomized clinical trials are required to estimate the effect of PRF on socket regeneration.
Bao et al. (2021)^27^	A-PRF showed a better effect among the three groups for the improvement of postoperative pain on the third and seventh days. L-PRF promoted soft tissue healing on the seventh day compared to the control. However, other comparisons did not show significant differences.	Limited results showed that applying A-PRF after third molar extraction reduced postoperative pain, and L-PRF promoted soft tissue healing on the seventh postoperative day. However, the positive impact on other postoperative sequelae was not confirmed. Further large-scale randomized controlled trials with uniformly standardized measuring methods are required to improve the analyses.
Bao et al. (2021)^28^	PRF showed significant positive effects on pain, edema, soft tissue healing, trismus, and alveolar osteitis. There was no statistically significant effect on bone healing.	Limited clinical evidence indicates that applying PRF after lower third molar extraction may reduce pain, edema, trismus, and dry socket alveolitis and promote soft tissue healing. However, the effect of PRF on bone healing requires further large-scale randomized controlled trials with uniform measuring criteria.
Barona-Dorado et al., (2014)^15^	Gürbüzer et al., 2008: No statistically significant differences. Rutkowski et al., 2010: PRP showed significantly smaller facial edema and higher bone density. Haraji et al., 2012: PRP showed significantly lower post operative pain, improved healing, and lower postoperative alveolar osteitis incidence.	The scientific evidence for using PRP in third-molar surgeries is scarce. Hence, randomized clinical trials are required before recommending the clinical application of PRP.
Canellas et al. (2017)^16^	PRF seemed to accelerate healing in mandibular third molar surgeries, reducing postoperative pain and edema. PRF significantly decreased alveolar osteitis prevalence.	Although further well-designed clinical trials with larger samples are required to allow definitive conclusions, PRF is a potentially suitable biomaterial.
Canellas et al. (2019)^25^	PRF showed significantly better bone regeneration for alveolar cleft reconstruction, decreased alveolar osteitis prevalence, and increased implant stability one week and one month after surgery.	PRF may present improved wound healing when used in oral surgical procedures. The most consistent indications of this systematic review are to preserve alveolar dimensions after extraction and to reduce alveolitis incidence after lower third molar surgery.
Costa et al. (2023)^32^	A-PRF and L-PRF resulted in lower pain levels on the first and third postoperative days compared to the blood clot. On the seventh postoperative day, pain was significantly lower for A-PRF, L-PRF, and PRP compared to the blood clot. There was a statistically smaller difference in the reduction of interincisal opening for A-PRF compared to L-PRF and the blood clot during the acute inflammatory period. A-PRF, L-PRF, PRP, and CGF demonstrated favorable outcomes in reducing edema in at least one evaluated period.	Given the low quality of evidence, A-PRF following lower third molar extractions demonstrated superior performance among the analyzed blood concentrates and appeared effective in reducing postoperative pain at all evaluated time points. Trismus decreased with the use of A-PRF during the peak inflammatory period.
Fujioka-Kobayashi et al. (2021)^17^	PRF significantly reduced the alveolar osteitis rate from 15.9% in the control group to 5.8% in the PRF group, representing an approximated three-fold reduction. PRF showed better results for soft tissue healing.	PRF decreases postoperative pain/morbidity, favors soft tissue healing, and presents lower complication rates of alveolar osteitis. Therefore, PRF represents a feasible auxiliary treatment after lower third molar removal.
He et al. (2017)^27^	PRF significantly relieved pain, reduced edema on the third postoperative day, and decreased alveolar osteitis incidence. There were no significant differences in swelling on the first postoperative and bone consolidation day.	The local application of PRF after lower third molar extraction is a valid method to relieve pain and edema at three postoperative days and reduce alveolar osteitis incidence. For patients subjected to complicated surgical extractions, PRF may be recommended for local socket applications.
Ramos et al. (2022)^30^	L-PRF showed better pocket depth, insertion level, and pain control (first and third days), but it was not associated with improved soft tissue healing on the seventh day. A-PRF presented lower analgesics intake. L-PRF and A-PRF controlled edema better but not trismus.	Using L-PRF and A-PRF allows better pain and edema c ontrol than standard PRF protocols, but none affected trismus. PRF and L-PRF protocols improved soft tissue healing but not to a statistically significant degree. However, they improved probe depth in the third month after the third molar surgery.
Ribeiro et al. (2024)^36^	Using L-PRF contributed to reduce postoperative pain and discomfort.	The L-PRF considerably reduced postoperative pain, edema, alveolar osteitis incidence, and infections following third molar removal surgery compared to patients not subjected to L-PRF application.
Snopek et al. (2022)^31^	PRF favored the reduction of alveolar osteitis frequency and had an uncertain effect on the level of postoperative pain, edema, and wound healing. Pain levels did not show statistical differences.	Considering the low number of included studies, the effect of PRF after lower third molar surgery could not be evaluated. Further studies should focus on a higher methodological quality to produce reliable and comparable study results.
Vitenson et al. (2022)^18^	A-PRF caused significantly lower pain scores than L-PRF and natural wound healing after two, three, and seven days. A-PRF had a significant effect on facial edema and trismus and some positive effects on soft tissue healing.	The included studies were characterized by considerable heterogeneity and confounding variables. Thus, the level of evidence seems inadequate for clinical recommendations according to the focus question.
Xiang et al. (2019)^26^	PRF relieved pain and edema and reduced alveolar osteitis incidence. There was no statistical difference for trismus and bone and soft tissue healing.	PRF reduces only some postoperative complications, but it does not prevent all of them. PRF significantly relieved pain and edema and reduced alveolar osteitis incidence after extracting an impacted lower third molar.
Zhu et al. (2021)^29^	PRF significantly reduced alveolar osteitis incidence and postoperative pain (first, third, and seventh days) compared to the controls.	Besides reducing alveolar osteitis incidence and postoperative pain after third molar surgery, it may also improve postoperative healing of soft tissues.

PRP: platelet-rich plasma; PRF: platelet-rich fibrin; A-PRF: advanced platelet-rich fibrin; L-PRF: leukocyte- and platelet-rich fibrin; CGF: concentrated growth factor. Source: Elaborated by the authors.

### Critical assessment of individual evidence sources

According to the AMSTAR-2^21^ analysis, only one study presented high methodological quality^33^, while six studies^15^^,^ 16 ^,^ 18 ^,^ 25 ^,^ 31 ^,^ 33 demonstrated low methodological quality, and the other systematic reviews were critically low^17^^,^ 23 ^,^ 24 ^,^ 26 ^–^ 30. Table 4 summarizes the responses of each systematic review to the AMSTAR-2^21^ questionnaire.

**Table 4:** Critical assessment of individual evidence sources of the included systematic reviews.

Authors, year	Questions																Methodological quality
	1	2**	3	4**	5	6	7**	8	9**	10	11	12	13**	14	15**	16	
Al-Hamed et al. (2017)^23^	N	N	N	PY	N	Y	Y	Y	Y	N	N	N	N	Y	N	Y	Critically low
Bao et al. (2021)^27^	Y	N	N	PY	Y	Y	N	PY	Y	N	Y	N	N	Y	N	Y	Critically low
Bao et al. (2021)^28^	Y	N	N	PY	N	Y	N	Y	Y	N	Y	N	N	Y	N	Y	Critically low
Barona-Dorado et al., (2014)^15^	Y	N	N	PY	N	N	Y	PY	PY	N	NM	NM	Y	Y	NM	N	Low
Canellas et al. (2017)^16^	Y	Y	N	Y	Y	N	Y	Y	Y	N	N	N	Y	Y	N	Y	Low
Canellas et al. (2019)^25^	Y	Y	N	Y	Y	N	Y	Y	Y	N	N	N	Y	Y	N	Y	Low
Costa et al. (2023)^32^	Y	Y	Y	Y	Y	Y	Y	Y	Y	Y	Y	Y	Y	Y	Y	Y	High
Fujioka-Kobayashi et al. (2021)^17^	Y	PY	N	Y	Y	Y	Y	Y	Y	N	Y	N	N	Y	N	Y	Critically low
He et al. (2017)^27^	N	N	N	PY	N	Y	N	Y	Y	N	Y	N	N	Y	Y	Y	Critically low
Ramos et al. (2022)^30^	Y	Y	N	PY	Y	Y	Y	Y	Y	N	Y	N	N	Y	N	Y	Critically low
Ribeiro et al. (2024)^36^	Y	Y	Y	Y	Y	N	N	Y	Y	Y	NM	NM	Y	N	NM	N	Low
Snopek et al. (2022)^31^	Y	Y	N	Y	Y	Y	Y	Y	Y	N	Y	Y	Y	Y	N	Y	Low
Vitenson et al. (2022)^18^	Y	Y	N	Y	Y	N	Y	Y	Y	N	Y	Y	Y	Y	N	Y	Low
Xiang et al. (2019)^26^	Y	N	N	PY	Y	Y	N	PY	Y	N	Y	N	N	Y	N	Y	Critically low
Zhu et al. (2021)^29^	Y	N	N	PY	Y	Y	N	Y	Y	N	Y	N	N	Y	N	Y	Critically low

1: “Did the research questions and inclusion criteria include the PICO components?”; 2: “Did the review report explicitly state that the methods were established before performing the review, and the report justify any significant deviations from the protocol?”; 3: “Did the review authors explain their study design selection for inclusion in the review?”; 4: “Did the review authors use a comprehensive literature search strategy?”; 5: “Did the review authors select the studies in pairs?”; 6: “Did the review authors extract the data in pairs?”; 7: “Did the review authors provide a list of excluded studies and justify the exclusions?”; 8: “Did the review authors describe the included studies with adequate details?”; 9: “Did the review authors use a satisfactory technique to assess the risk of bias (RoB) in individual studies included in the review?”; 10: “Did the review authors inform the funding sources of the studies included in the review?”; 11: “In case there was a meta-analysis, did the review authors use adequate methods to statistically combine the results?”; 12: “In case there was a meta-analysis, did the review authors assess the potential impact of RoB of individual studies on the results of the meta-analysis or other evidence synthesis?”; 13: “Did the review authors consider RoB in individual studies when interpreting/discussing the review results?”; 14: “Did the review authors provide a satisfactory explanation and discuss any heterogeneity verified in the review results?”; 15: “In case there was a quantitative synthesis, did the review authors perform an adequate investigation on the publication bias (small study bias) and discuss its probable impact on review results?”; 16: “Did the review authors report any potential conflict of interest sources, including any funding received for conducting the review?”; Y: yes; PY: partially yes; N: no; NM: no meta-analysis performed;

** critical domain.

Source: Elaborated by the authors based on the AMSTAR-2 tool.

### Risk of individual bias of the eligible studies

Only two systematic reviews^31^^,^ 32 showed low risk, and the others had a high risk of bias^15^^–^ 18 ^,^ 23 ^–^ 30 ^,^ 33. There were relevant concerns regarding the risk of bias, including:

- The absence of a previous study protocol registration;
- The absence of a comprehensive electronic search in grey literature databases and with additional strategies;
- The absence of a definite mention of at least two eligibility reviewers in the methodological steps of the systematic review;
- The absence of sensitivity analyses;
- Primary studies with a high risk of bias;
- Protocol deviations;
- Data interpretation focused on statistically different results.

**Figure 3:**
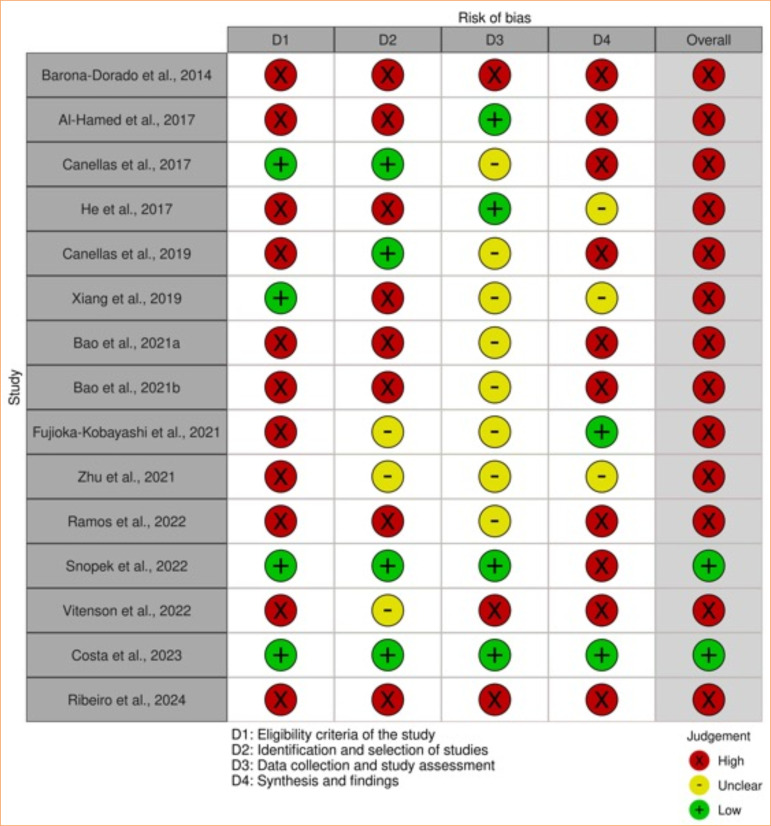
Individual risk of bias of the eligible studies.

Source: Elaborated by the authors.

Figure 3 summarizes the overall findings of the risk of bias assessment.

## Discussion

The present overview analyzed the current knowledge on the performance of blood concentrates in handling sequelae after lower third molar extractions with the evidence available in systematic reviews. Considering the surgical nature and complexity, third molar removal may be associated with postoperative sequelae, such as pain, edema, mouth-opening difficulties, and alveolar osteitis^2^. The healing process involves a highly coordinated sequence of biochemical, physiological, cellular, and molecular responses to restore tissue integrity. Blood concentrates have been extensively used to improve tissue repair and modulate the postoperative inflammatory process^34^^,^ 35.

Only two systematic reviews^15^^,^ 32 evaluated PRP, and the evidence available for using PRP in lower third molar extractions is scarce. Barona-Dorado et al.^15^ included three primary studies^36^^–^ 38 in the cited systematic review^15^, showing conflicting results regarding the efficacy of PRP in controlling pain, mouth opening, and alveolar osteitis. Two primary studies^37^^,^ 38 did not present methodological flaws in the clinical trials but showed result interpretation biases. One of the articles^38^ affirmed that PRP significantly reduced pain levels but did not provide any value (mean and standard deviation). It did not determine the method for diagnosing alveolar osteitis and analyzed the variables jointly between upper and lower third molar sockets either. The other study^37^ did not provide objective data for the analyzed variables and had a small sample. It is worth noting that this included systematic review^15^ also presents methodological performance biases. The risk of bias analyzed in the ROBIS tool^22^ showed that, despite determining the study objective, there was no review question. Moreover, the review neither performed a manual search in the references of the included primary studies nor mentioned the participation of two reviewers during data collection and the risk of bias classification in said studies.

Costa et al.^32^ selected nine studies assessing PRP performance^39^^–^ 47. Five of these articles^39^^,^ 40 ^,^ 43 ^,^ 45 ^,^ 46 reported statistically favorable outcomes for the group treated with the blood concentrate, four^40^^,^ 42 ^,^ 43 ^,^ 45 concluded that PRP significantly reduced edema compared to blood clots, and three^43^^,^ 45 ^,^ 46 presented a lower occurrence of trismus. Although the study by Costa et al.^32^ had a high methodological quality according to AMSTAR 2^21^ and a low risk of bias according to ROBIS^22^, the results require careful interpretation, as the primary studies assessing PRP performance exhibited a moderate to high risk of bias.

Conflicting findings also appeared for the role of L-PRF in controlling postoperative sequelae after lower third molar removal. Most included systematic reviews presented favorable conclusions regarding the efficacy of blood concentrates^16^^,^ 17 ^,^ 24 ^,^ 26 ^–^ 30 ^,^ 32. However, it is worth noting that three of these investigations^16^^,^ 27 ^,^ 28 ^,^ 32 also highlighted outcome limitations and the need for randomized clinical trials with larger samples and a more homogeneous methodology for a higher comparison effect. Except for the study by Costa et al.^32^, the risk of bias analysis with the ROBIS tool^22^ revealed that all reviews present at least a few methodological flaws concerning the lack of reference to a registration protocol^24^^,^ 26 ^–^ 29, language limitations^17^^,^ 26 ^,^ 30, or the absence of a definite mention of at least two eligibility reviewers during study selection^24^^,^ 28, data extraction^16^, and risk of bias assessment^24^^,^ 26 ^,^ 28 of primary studies. Some methodological flaws, such as the lack of a previous registration protocol, may promote imprecise conclusions, as it is hard to know whether these criteria were established in advance and guided the reviewers’ steps during the review or were determined or modified in the review process^21^^,^ 22. Therefore, the findings of the referred systematic reviews must be carefully interpreted considering the identified methodological limitations to prevent precipitated or imprecise conclusions.

Some systematic reviews included in the present overview did not have sufficient evidence to indicate the use of PRF after lower third molar extraction2^3^^,^ 25 ^,^ 31. Including patients with lower third molars at varying degrees of inclusion/impaction in a single sample may have introduced biases in clinical trial results^23^. Additionally, surgical difficulty presents a statistically significant relationship with the intensity of postoperative sequelae^48^^,^ 49. It is worth noting that one systematic review^23^ presented a high risk of methodological bias due to the lack of reference to previous study protocol registration, the absence of a comprehensive electronic search in grey literature databases, the absence of a definite mention of at least two eligibility reviewers during study selection and risk of bias assessment, and the absence of sensitivity analyses. Another systematic review with a high risk of bias^25^ needs careful interpretation because, except for one of its primary studies^50^, the others did not present randomized clinical trial registrations, and the statistically insignificant results might have been hidden in the final publication, introducing result interpretation biases. Also, the primary studies of one of the included systematic reviews^31^ were split mouth and double-blind clinical trials, and the statistical methods used in the meta-analysis allowed a reliable interpretation of results. The statistical methods of the meta-analysis allowed a reliable interpretation of results, but the methodological heterogeneity in measuring edema and insufficient information on alveolar osteitis limited evidence availability. Consequently, a meta-analysis for postoperative pain was only feasible in three randomized clinical trials from the same systematic review^31^, highlighting the limitations of available evidence.

Our overview showed that A-PRF reduced pain in the first^30^^,^ 32, second^18^, third^18^^,^ 27 ^,^ 32, and seventh^32^ postoperative days. As for trismus and edema, A-PRF promoted higher reduction during and after the third postoperative day^18^^,^ 32. These results seem debatable because the release of growth factors through A-PRF occurs for 10 days, and the physiological inflammatory response appears immediately after the tissue lesion^51^^,^ 52. Thus, these data should be carefully interpreted because randomized clinical trials presented methodological heterogeneity, potential result interpretation biases18, methodological flaws for not mentioning the predefined registration protocol^28^, restrictions on publication language18, or the absence of two reviewers during study selection and data extraction of the primary studies^28^.

While systematic reviews are essential for evidence-based decision-making, accepting their results without a critical assessment may provide improper conclusions^21^. A critical evaluation should involve deep knowledge of the referred therapy and the physical and biological blood concentrate properties, indications, advantages, and limitations. Therefore, the limitations of this overview stem from the high heterogeneity and risk of bias of the included systematic reviews and their primary studies, complicating the establishment of definitive evidence. However, it is worth noting that the data for clinical decision-making on blood concentrate use should be carefully interpreted, as a detailed analysis showed relevant methodological flaws in apparently robust systematic reviews on the topic.

Additionally, systematic reviews of high-quality randomized controlled trials are essential to provide accurate evidence on this topic. In this aspect, high-quality randomized controlled trial should present an adequate randomization process, sample size calculation, blinding of operator, patient and outcome assessor, an adequate follow-up period, and protocol registration.

## Conclusion

Biological factors inherent to blood concentrates are promising for optimizing tissue repair. However, the current evidence from systematic reviews remains regarding the efficacy of these products in managing sequelae after lower third molar extractions.

Randomized clinical trials with methodological standardization are first required for considering systematic reviews as adequate references for evidence-based decision-making. Moreover, systematic reviews must be performed according to scientifically established guidelines to minimize biases and ensure methodological rigor.

## Data Availability

All data sets were generated or analyzed in the current study.
